# Development of a cost-effective, multifunctional SNP panel and analysis workflow for Wolf monitoring in Finland

**DOI:** 10.1038/s41598-025-24502-w

**Published:** 2025-11-19

**Authors:** Jenni Harmoinen, Mia Valtonen, Daniel Fischer, Terhi Iso-Touru, Mikael Åkesson, Anne-Maarit Heikkinen, Katja Holmala, Ilpo Kojola, Elina Salmela, Maris Hindrikson, Urmas Saarma, Hannes Lohi, Laura Kvist, Jouni Aspi, Helena Johansson

**Affiliations:** 1 Wildlife Ecology Group, Natural Resources Institute Finland, Helsinki, Finland; 2 Ecology and Genetics Research Unit, University of Oulu, Oulu, Finland; 3 Applied Statistical Methods, Natural Resources Institute Finland, Jokioinen, Finland; 4 Genomics and Breeding group, Natural Resources Institute Finland, Jokioinen, Finland; 5 Department of Ecology, Grimsö Wildlife Research Station, Swedish University of Agricultural Sciences, Riddarhyttan, 739 93 Sweden; 6 Natural Resources Institute Finland, Helsinki, Finland; 7 Wildlife Ecology Group, Natural Resources Institute Finland, Rovaniemi, Finland; 8 Organismal and Evolutionary Biology Research Programme (OEB), University of Helsinki, Helsinki, Finland; 9 Department of Biology, University of Turku, Turku, Finland; 10 Department of Zoology, Institute of Ecology and Earth Sciences, University of Tartu, Tartu, Estonia; 11 Department of Veterinary Biosciences, University of Helsinki, Helsinki, Finland; 12 Department of Medical and Clinical Genetics, University of Helsinki, Helsinki, Finland; 13 Folkhälsan Research Center, Helsinki, Finland

**Keywords:** Wildlife management, Microfluidic array, Non-invasive samples, Single-nucleotide polymorphism, Grey wolf, Microsatellites, Biological techniques, Ecology, Evolution, Genetics, Ecology

## Abstract

Genetic monitoring is an essential tool for managing threatened or commercially valuable wildlife species. Ideally, genetic monitoring relies on straightforward protocols for genotyping and data handling, accommodates varying sample volumes, applies to non-invasive samples such as scat and hair, produces reliable and repeatable genotypes, and is cost-effective. Moreover, the marker panel itself should be tailored to specific species and/or population management information needs. We describe the development of a microarray-based 96-SNP panel specifically designed for non-invasive genetic monitoring of the Finnish wolf population. The panel was developed to meet four specific needs for this population: identification of wolf genotypes from the non-target species (fox, raccoon dog, golden jackal), sexing, individual identification, and kinship resolution. We demonstrate the utility of the SNP panel using a subset of the genetic monitoring data collected in 2022–2023, including an overview of the data analysis workflow. The development and successful application of this SNP panel, along with a comparison with similar studies in the field, provide valuable insights for researchers and conservationists aiming to implement similar strategies for other species.

**Supplementary Information:**

The online version contains supplementary material available at 10.1038/s41598-025-24502-w.

## Introduction

Population monitoring is essential for wildlife management, involving continuous spatio-temporal efforts to assess population trends over time, providing a more accurate understanding of population dynamics than one-time assessments. In Finland, the grey wolf (*Canis lupus*) population is closely monitored by the Natural Resources Institute Finland (Luke). This monitoring relies on non-invasive genetic sampling - primarily through scats - to track the population’s status and structure. These efforts include identifying breeding units for the annual census estimates, which require data on the number of unique individuals, their sex, and kinship relationships^1,2^.

The Finnish wolf population is currently estimated at approximately 400 individuals, with its main distribution in western Finland^3^. The wolf is classified as endangered in Finland according to the IUCN criteria^4^ and is therefore protected under current EU legislation. However, the species is also considered a game species under Finnish legislation (Hunting Act 615/1993), and lethal removal of certain individuals is permitted with special licenses, usually issued due to wolf-livestock conflicts. Northern Finland is dedicated to reindeer husbandry, which is widely practiced by the indigenous Sámi people. In this area, wolf hunting is permitted to prevent damage to the practice. Consequently, wolf territories do not become established in the area. In regions located outside of the reindeer husbandry area, a maximum number of licenses are issued annually by the Ministry of Agriculture and Forestry, while no fixed maximum is set for the reindeer husbandry area.

Since 2014, Luke’s wolf monitoring program has included the collection of non-invasive DNA samples, primarily sourced through citizen science, with professional sampling conducted in areas that would otherwise remain unsampled. Sampling has focused on areas where wolves have been observed, with geographic coverage expanding over the years to encompass all potential territories. When scat is collected by volunteers, it is not uncommon for samples from non-target canid species such as dogs (*Canis familiaris*), red foxes (*Vulpes vulpes*), and raccoon dogs (*Nyctereutes procyonoides*) to be included. This necessitates the identification and exclusion of non-target species from the data. Additionally, the golden jackal (*Canis aureus*), a newcomer to the region, has become a focal point of interest for detection^5–7^.

Genetic monitoring has traditionally relied on microsatellite markers, which are commonly used in wildlife management due to their reliability, high information content, and straightforward genotyping process with rapid turnaround from sample to genotype^8^. While microsatellites require little technical expertise for data handling, standardizing results among labs is challenging due to variations in scoring procedures and instrument performance^9,10^. Microsatellites can be used to genotype non-invasive low-quality DNA samples, such as scat and hair, which are commonly used in genetic monitoring (e.g.,^11–13^). However, their usage necessitates a multi-tube approach, resulting in significant costs^14^. During the annual Finnish wolf monitoring in 2021–2022, over a thousand scat samples were collected. The high sample volume incurred high genotyping costs, highlighting the need for a more cost-efficient genotyping method that can handle large sample volumes without compromising data quality.

The development of cost-effective, high-quality Next Generation Sequencing (NGS) technologies offers a promising alternative to microsatellites in genetic monitoring^8,10,13,15^. A targeted, highly reduced set of informative SNP markers is selected from NGS or array data, aiming to develop protocols for repeatable genotyping. Some genotyping protocols rely directly on NGS, such as GT-seq^16^ or Genotyping-by-Sequencing^17^. However, establishing analysis pipelines requires bioinformatic expertise and, ideally, prior experience with laboratory protocols^ (8^ and references therein). Different sample volumes can be managed through various approaches, such as allocating sequencing flow cells across multiple projects, employing commercial sequencing services, or utilizing a range of sequencers with varying output capacities. However, these approaches do not offer the same level of simplicity or accessibility as microsatellite genotyping.

An SNP genotyping option that closely resembles the microsatellite genotyping experience is the use of microfluidic arrays. Several platforms have been developed by biotechnology companies, including Standard Biotools (formerly Fluidigm), Agena Bioscience (MassARRAY), and Amplifluor^8^. For example, the laboratory workflow for the Standard Biotools platforms is comparable in complexity to microsatellite genotyping and accommodates high throughput as well as some flexibility in sample volumes through the use of 96 SNPs by 96 sample arrays. Genotype scoring is straightforward and standardized with freely available software. Microfluidic arrays typically exhibit greater sensitivity than microsatellite genotyping, often yielding more and higher quality genotypes even from low-quality non-invasive samples^8,18–20^. Several different SNP panels have now been developed and described for microfluidic array platforms for a range of species and purposes, including carnivores [e.g.^18,]^^19,]^^21^,^22^], ungulates [e.g.^23^], birds [e.g.^28^], and fish [e.g.^25^].

In this study, we developed a cost-effective, multifunctional SNP panel and genotyping workflow specifically tailored to the needs of the Finnish wolf population. This panel was designed to (1) allow efficient differentiation of wolf samples from foxes, golden jackals and raccoon dogs, and flag samples potentially originating from dogs, or wolf-dog hybrids (2) include sex-chromosomal markers for sex identification, and (3) select highly heterozygous markers for reliable individual identification and (4) kinship analysis. We demonstrate how this SNP panel and microfluidic array workflow can provide accurate, repeatable data on individual wolves, enabling more effective and economical monitoring of the Finnish wolf population. Finally, we highlight how the new workflow is currently integrated into Luke’s annual monitoring practices, enhancing Finland’s capacity for data-driven wolf management.

## Materials and methods

### Identifying markers from the Illumina CanineHD BeadChip dataset

We utilized an Illumina CanineHD Whole-Genome BeadChip microarray (Illumina, Inc. San Diego, California, USA) dataset, which includes 173,662 SNPs. The SNPs in the Illumina CanineHD Whole-Genome BeadChip microarray were selected to maximize variation between modern dog breeds. Wolves are genetically close to dogs so wolves genotype successfully with this array, however the SNPs are inherently biased toward dog-specific variation and less representative for non-dog canids^26^. The dataset used here comprised 37 Finnish, 23 Russian, 10 Estonian, and 24 Scandinavian wolf samples, along with six Estonian golden jackal samples and 274 dogs representing 55 dog breeds (~ 5 individuals per breed), which also included two wolf-dog breeds: Saarloos Wolfdog (*n* = 5) and Czechoslovakian Wolfdog (*n* = 5). The Finnish wolf samples were almost exclusively collected from Eastern Finland and the reindeer husbandry area because that is where most of the derogation licenses are issued. All wolf and golden jackal samples, except two Finnish samples (hair and saliva) and one Scandinavian sample (blood), were tissue samples. All samples from dogs were blood. All samples, except for 19 Scandinavian wolves and six golden jackals, were previously employed in a wolf-dog hybridization study^22^.

Individuals with a genotyping success rate below 0.85 (five Russian wolves) and SNPs with a call rate below 90% were excluded from the dataset. Linkage disequilibrium (LD) was calculated in a window of 50 SNPs, shifting in steps of 5 SNPs, pruning one SNP from pairs with high LD (r^2^ > 0.2) using PLINK 1.9^27,28^.

The data from all wolves and golden jackals were used to identify SNPs that distinguish between the two species. F_ST_ values were calculated for each locus by treating wolves and jackals as separate populations, using the diveRsity package^29^ in R 3.6.3. The SNPs with the highest F_ST_ (F_ST_ = 1) between the species were extracted, and five candidates were taken for panel testing. Additionally, five markers were selected from a wolf-dog hybrid detection SNP panel developed by Harmoinen et al.^22^ to enhance the ability to identify dog samples within the wolf monitoring data.

To identify markers with high minor allele frequencies (MAF), only Finnish wolf data was used, filtering out SNPs deviating from Hardy-Weinberg equilibrium (HWE, *P* < 0.001) using PLINK. Subsequently, MAFs were calculated for the remaining SNPs. Out of the 990 SNPs with the highest possible MAF (0.5), 145 were randomly chosen for testing purposes. The samples genotyped with the Illumina CanineHD BeadChip were biased towards eastern Finland (see above) with low representation of the inbred packs in southwestern Finland (see below). Therefore, SNPs with the highest possible MAF were targeted, assuming that it would increase the chance of obtaining markers with MAFs in the range 0.3–0.5 (suitable for kin discrimination) during optimization on samples obtained from the whole of Finland.

The X-chromosomal SNPs were extracted and those located in the pseudoautosomal region (PAR) of the X chromosome were removed, as SNPs in the PAR region undergo recombination between the sex chromosomes^30^. Subsequently, females and males were separated into their respective datasets and the minor allele frequency was calculated for both datasets. For the female dataset, 15 SNPs that exhibited the highest minor allele frequencies (MAF), ranging from 0.125 to 0.333, were selected. Conversely, for the male dataset, the SNPs with the lowest MAF (< 0.005) were chosen, since males, possessing only one X chromosome, are expected to show homozygosity at these loci, whereas females, with two copies of the X chromosome, are more likely to be heterozygous.

### Test of markers used in the Scandinavian wolf monitoring SNP panel

Forty-eight SNPs from the Scandinavian wolf monitoring panel (SLU, NINA, unpublished) with the highest MAF were selected for testing. Additionally, for male sex determination, a Y-chromosomal marker from the same panel was included, which has already been demonstrated to work well for this purpose in the annual Scandinavian wolf monitoring (pers. com. Mikael Åkesson).

### Primer design

Primer design was performed by Standard Biotools, using their D3 assay design tool (https://d3.standardbio.com*)* for the target SNPs and 200 bp of flanking sequence. For each SNP, one specific target amplification primer (STA), two allele-specific primers (ASPs), and one reverse locus-specific primer (LSP) were designed. In total, primers were ordered for 219 SNPs, including five for discriminating wolves and dogs, five for discriminating wolves and golden jackals, 193 high-MAF markers (including 48 from the Scandinavian SNP panel), 15 X-chromosomal markers, and one Y chromosomal marker.

### Microfluidic genotyping and final selection of markers

Several classes of samples were selected to test the diverse marker categories. For species discrimination, tissue samples from various species were used: two golden jackals, five red foxes, three raccoon dogs, two brown bears (*Ursus arctos*), two Eurasian lynxes (*Lynx lynx*), and two moose (*Alces alces*). For the initial sex-chromosomal and high-MAF marker testing, 79 wolf tissue samples were used. All markers from all categories were further tested with 15 scat, 12 urine, 18 saliva, and two blood samples from wolves. The different types of sample material from wolves were used to ensure amplification success across a range of sample qualities. The different sample materials contain varying amounts and purity of DNA, which can result in marker dropouts^19^. Therefore, all scat samples, often with the lowest or most degraded DNA content, were genotyped in duplicate^31^. A positive control (tissue sample with a known genotype) and a negative control (no template in the reaction) were included in each run.

The final panel was genotyped on a set of 36 unrelated individuals selected from across Finland to test for deviations to the Hardy-Weinberg equilibrium and linkage disequilibrium and to calculate final population-representative minimum allele frequencies (MAF) and probability of identity (PID).

For genotyping, 96.96 Dynamic Array™ IFC plates were utilized (Integrated Fluidic Circuit plates, Standard BioTools Inc, formerly Fluidigm) with a pre-amplification protocol developed for Scandinavian wolves (SLU, NINA unpublished), as follows: 5 µl reactions with 2.6 µl of Qiagen multiplex master mix and 2 µl DNA (undiluted), using 28 cycles for pre-amplification (STA) and diluting 1 µl of the PCR product in 8 µl of ddH_2_O. The IFCs were amplified on the (Standard BioTools Inc) Juno platform, and the allele-specific fluorescence was measured by an endpoint reader (Biomark EP1 reader). The raw genotype data was analyzed with the SNP Genotyping Analysis Software v. 4.5.1 (https://www.standardbio.com/products/software*)*, and scores were manually validated from the automatically scored scatter plots.

Markers were initially scored based on readability, and only well-amplifying markers with clear genotype clusters were selected. Assay reference libraries were constructed from the successful runs and incorporated in the subsequent scoring to facilitate genotype calling for the selected markers. Genotypes for species and sex markers were verified to ensure they conformed to the expected frequencies. MAFs were calculated, and significant deviations from LD (R and R^2^ *p* < 0.05) and HWE (*p* < 0.05) were tested using PLINK.

The marker set underwent refinement through several iterations of genotyping on different combinations of samples, following the analyses described above, until a final panel was selected. The final panel comprised two jackal-wolf markers, three dog-wolf markers, nine X-chromosomal markers, one Y-chromosomal marker, and 81 high-MAF markers (including 21 from the Scandinavian panel) (Supplementary Tables file: Tables 1 and 2).

**Table 1:** Tested and rejected markers. Some markers failed in more than one category.

Marker type	High MAF (Scand)	X chrom.	Y chrom.	Wolf-dog	Wolf-jackal
Number of markers	193 (48)	15	1	5	5
No amplification	12 (1)				
Low amplification	9 (5)	1			1
NTC up	4 (1)				
Readability	32 (6)	3		1	1
Low MAF	23 (7)				
Deviation from HW	20 (5)				
LD	5 (1)	1			

No amplification, none of the samples amplified; Low amplification: only part of the tested samples amplified, NTC up, non-template control showed significant fluorescence; Readability, genotype clusters were not distinct; Low MAF, too low MAF (< 0.3); Deviation from HW, significant deviation from HWE (*p* < 0.05); LD, significant deviations from LD (R and R^2^ *p* < 0.05).

**Table 2:** Average amplification success of non-target species with the SNP panel. Dogs amplified equally well as wolf samples (data not shown).

Species	Sample size	Average genotyping success (%)
Golden jackal (*Canis aureus)*	2	97
Red fox (*Vulpes vulpes*)	5	83
Raccoon dog (*Nyctereutes procyonoides)*	3	87
Brown bear (*Ursus arctos)*	2	14
Eurasian lynx (*Lynx lynx)*	2	10
Moose (*Alces alce*s)	2	8

Some test runs were conducted following the manufacturer’s manual with Juno 96.96 Genotyping IFC plates. In this setup, the pre-PCR step (STA) is incorporated on the same plate with PCR involving fluorescently labelled allele-specific markers . However, it was observed that the DNA concentrations in the scat samples were too low or the DNA was too degraded for this type of IFC. Consequently, the genotyping rate appeared lower (60%) than with the 96.96 Dynamic Arrays. Therefore, further testing was discontinued, and optimization efforts continued with the 96.96 Dynamic Arrays.

### Performance of the final panel

#### Error rates

Wolf tissue samples with known Illumina CanineHD BeadChip genotypes (*N* = 15) were genotyped using the developed SNP panel, and the genotypes were compared for error rate estimation purposes. All markers, except the Y marker not included in the Illumina CanineHD BeadChip, were utilized (95 markers). To estimate genotyping error rates, the genotype based on the Illumina chip was assumed to be accurate. Only loci with non-missing Illumina chip genotypes were considered for the comparison. An allelic mismatch was documented in cases where the Illumina genotype was heterozygous and the designed SNP panel showed homozygotes, or vice versa. Mismatches were also noted if the genotypes were opposite homozygotes. Missing genotypes were recorded based on missing genotypes with the designed SNP panel, regardless of Illumina genotypes.

#### Identification of wolf genotypes

To test the data and develop the analysis for accurately identifying wolf genotypes from non-target species, the samples from several different species genotyped above were analyzed alongside the Illumina CanineHD BeadChip data of 37 Finnish wolves, 6 golden jackals, and 274 dogs. This analysis was performed using the *snmf* function and Principal Component Analysis (PCA) within the R package LEA^32^. Only autosomal markers of the panel (86 SNPs) were included.

The sNMF analysis was conducted with *K* (the number of populations) 1–10 with 10 replicates per *K*. The most likely number of populations (*K*) was determined by plotting the cross-entropy values for each *K* and examining both the greatest drop in entropy between subsequent *K*-values, and the *K* with the lowest entropy value. Individual ancestry coefficients (*Q*) were extracted from the best run with the most likely *K* and plotted.

**Fig. 1:**
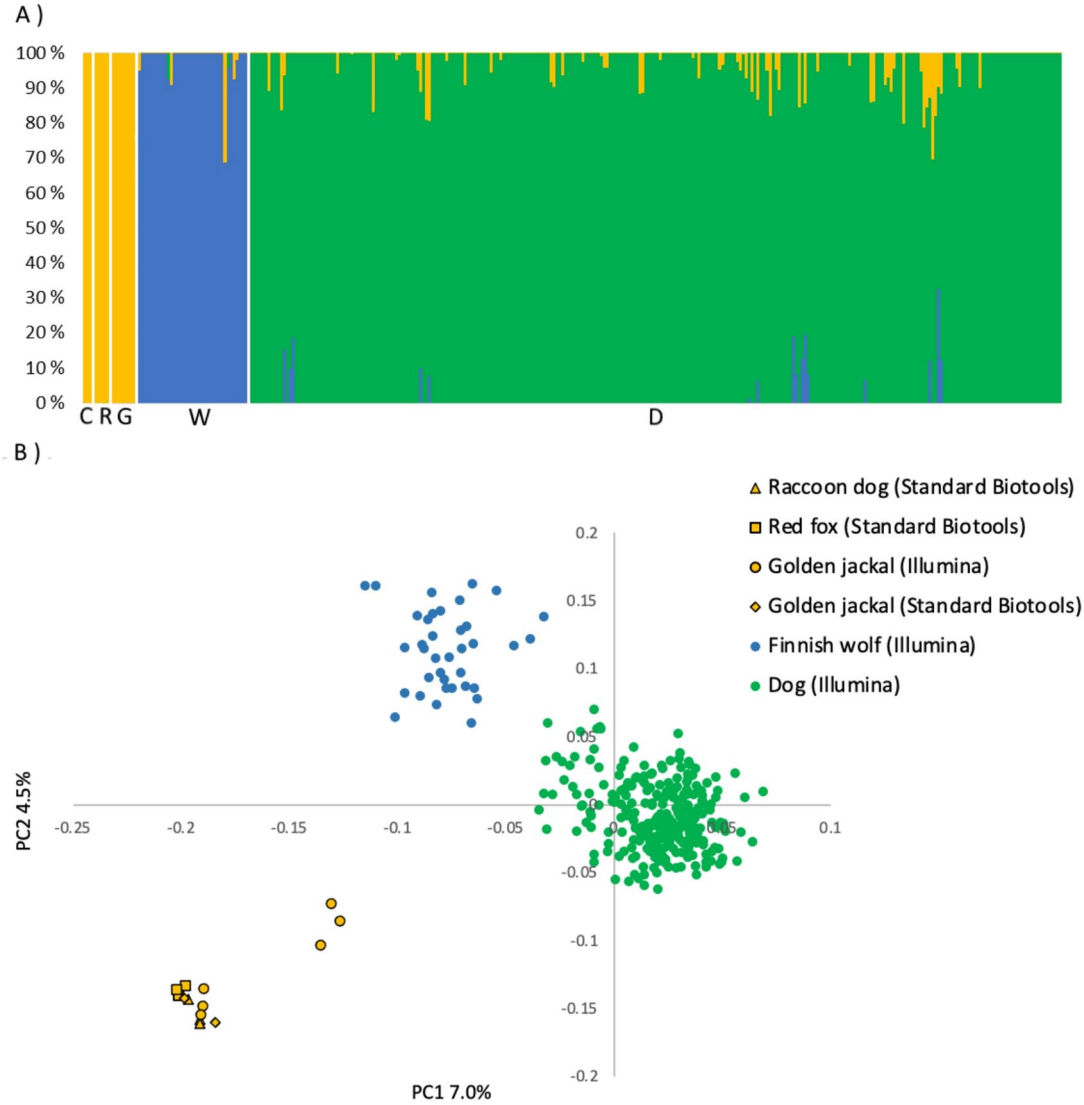
(**A**) The sNMF analysis for different species with the most likely number of clusters *K* = 3 using the 86 autosomal markers from the SNP panel. Species are marked with initials: C for raccoon dog, R for red fox, G for golden jackal, W for wolf, and D for dog. (**B**) PCA analysis of the same data.

Results from sNMF analysis were used to construct reference groups for the two RUBIAS assignment analyses^33^. First, dogs and wolves with sNMF Q-values above 0.85 (*N* = 299) were pooled into one reference group and all jackal, raccoon dog and fox genotypes were pooled into a second reference group. The Q-value cutoff was based on manual inspection of the Q-value distributions^34^, aiming to maximize assignment certainty while retaining within-species variation (Fig. 1). Individuals with Q-value below this threshold were excluded. The second assignment approach included extracting reference populations for only wolves and dogs from the sNMF analysis above (Q-values above 0.85), followed by a RUBIAS analysis as outlined above. The performance of these reference populations was assessed by self-assignment and a leave-one-out analysis in RUBIAS with default settings.

#### Validation of sex markers

To validate the sex markers, Illumina genotyped samples used in pedigree analyses (*N* = 67) and a subset of samples from the Finnish wolf population where sex was known (*N* = 39) were employed (in total *N* = 87). Genetic sexing was conducted using the *--check-sex* function in PLINK.

#### HWE, MAF, linkage disequilibrium tests and probability of identity

Loci deviating from HWE and MAF were calculated using PLINK on unrelated individuals collected across Finland. The probability of identity (PID) and probability of identity between siblings (PIDsib) were computed using GenAlEx 6.5^35^, utilizing the same individuals. All SNPs were included except the Y marker and one marker (TIGRP2P103821_rs9012738) with excessive missing data. A threshold of PID < 0.0001 was selected as a sufficiently low PID for wild populations^19,36^.

#### Pedigree analyses of inbred wolf family line

Inbred wolf packs comprising 67 individuals from southwestern Finland were genotyped to evaluate the panel’s resolution in sorting out the pedigrees of these packs, whose histories were known based on the field data and further supported by microsatellite data. Pedigree-based inbreeding coefficients were calculated, and pedigrees were drawn using Pedigraph v. 2.4^37^.

Eighty-one SNPs from the developed panel (excluding sex markers and species-specific markers) and 17 microsatellites from Mäntyniemi et al.^38^ were used in separate COLONY 2.0.6.6^39^ analyses. Results were compared for samples overlapping between the datasets (see Supplementary Material, Fig. 2). All individuals were considered as potential offspring, all females as potential mothers, and all males as potential fathers. Individuals with unknown sex were included as potential mothers and fathers. The analyses were performed three times for both marker sets (replicates) assuming 0.01 error rates, with a 0.9 probability that parents are in the data. The analysis allowed polygamy and inbreeding and employed the full likelihood model with very long runs and high precision. Offspring-parent relationships with a completely missing genotype in either marker type (due to limited sample availability or quality) were removed from the comparison.

**Fig. 2:**
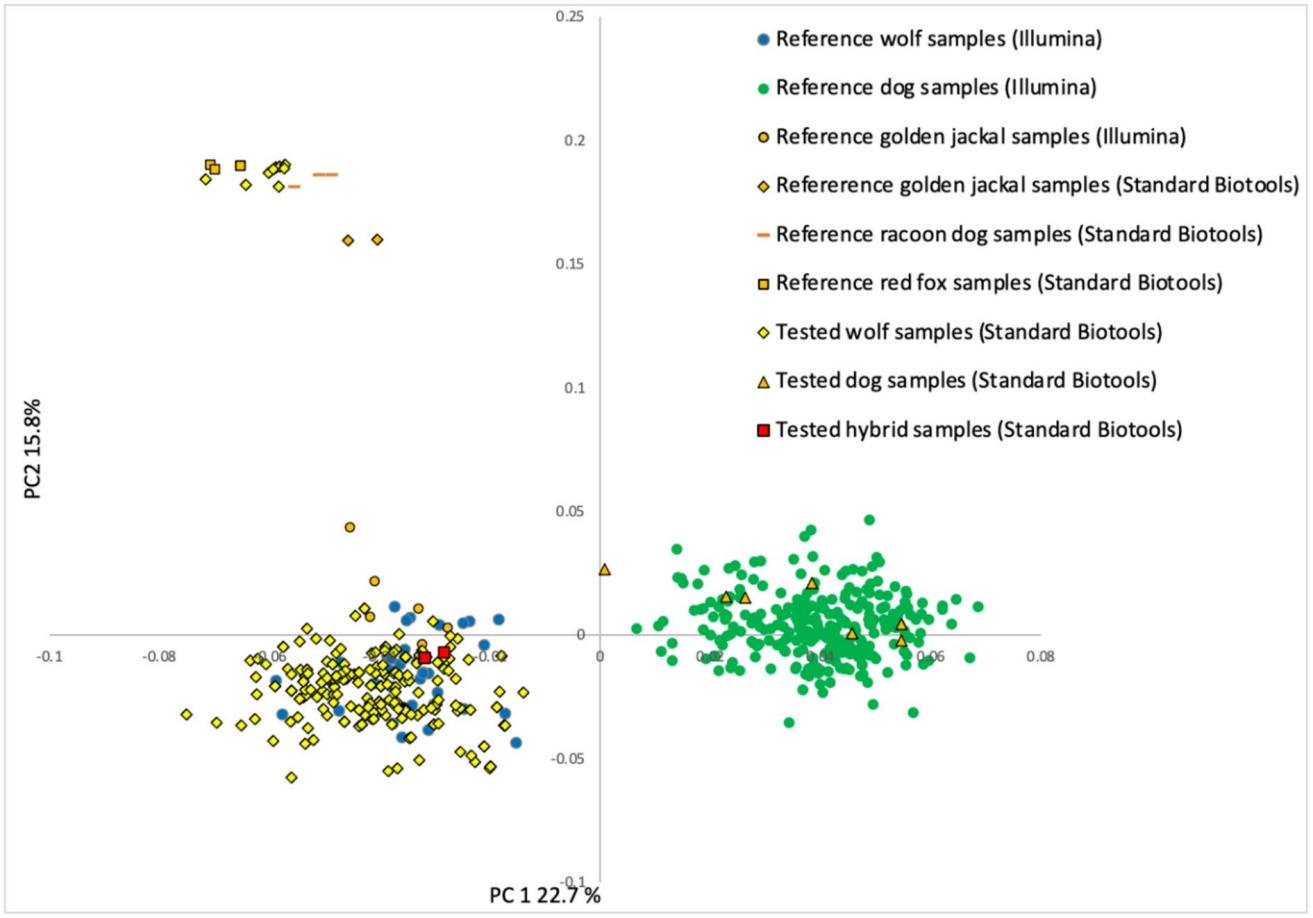
A principal component analysis (PCA) for 233 samples representing a subset of wolf monitoring data from 2022–2023, analyzed alongside reference samples of wolves and dogs, hybrids, golden jackals, foxes and raccoon dogs.

#### Using the SNP panel in regular wolf monitoring

The final SNP panel was employed during the wolf monitoring season 2022–2023. A subset of 282 samples from this monitoring season was utilized to illustrate the performance of the SNP panel in the wolf monitoring program in Finland. The sample subset comprised various sample types: 188 scat, 74 urine, 16 tissue, and 4 hair samples, which originated from wolves, dogs, dog-wolf hybrids, and foxes. The complete set of analyses is described in Supplementary Materials file Sect. 1.

## Results

### Panel development

Out of the 219 SNP markers that were tested, a subset was discarded for the following reasons: 21 markers failed in amplification or had a low amplification rate in the tested primer combination, four markers showed amplification in the non-template control more than once, 32 markers were rejected due to poor readability of genotype clusters (e.g. clusters were too close to each other, weirdly placed or one cluster was missing), 23 high MAF markers exhibited a lower than acceptable MAF when tested with newly genotyped samples, 20 markers deviated from HWE, and five markers were in LD with another marker. Some markers were excluded due to more than one category of failure. The markers included in the final panel are listed in Supplementary Tables file: Table 1, along with assay IDs and primer sequences.

### Error rate

Genotypes obtained with the developed SNP panel were consistent with the known genotypes. No allelic differences were detected out of the 1425 pairwise comparisons (15 samples x 95 markers). The average amplification success rate was 97% across both samples and loci, primarily affected by one X-chromosomal marker (BICF2P1098274) failing completely. It is important to note that this result applied only to tissue samples, as we did not have Illumina-generated genotypes from other types of sample material.

### Species identification

Species within the same taxonomic family as wolves—such as dog (which amplified equally well as wolves, data not shown), golden jackal, red fox, and raccoon dog—were successfully amplified with the SNP panel, and their genotypes passed the data filtering process (Table 2). However, more distantly related species—brown bear, Eurasian lynx, and moose—exhibited significantly lower amplification success rates and did not pass the analysis stage.

The SNP panel demonstrated high resolution in distinguishing between species that passed the data filtering (Fig. 1). The largest drop in cross-entropy from the between sequential K-values occurred between *K* = 2 and *K* = 3 in the sNMF analysis, suggesting *K* = 3 to be most likely, whereas the lowest cross-entropy value was found for *K* = 4 (Supplementary Material file: Fig. 1). Individual ancestry coefficients (Q-values) were extracted from the best run for K = 3 and K = 4 and examined. For *K* = 3, raccoon dog, red fox, and golden jackal genotypes clustered together, and all had *Q*-values above 0.9 (Fig. 1A), suggesting very robust clustering. All dog genotypes, including wolf-dog breeds, formed another distinct cluster with *Q-*values above 0.66, with only 4% of *Q-*values below 0.9 (Supplementary Tables file: Table 3). Similarly, all wolf genotypes were assigned to their own cluster, with *Q*-values above 0.77, whereas 8% had *Q*-values below 0.9 (Supplementary Tables file: Table 3). For *K* = 4, results for golden jackals, foxes, raccoon dogs and wolves were highly similar, however, the dogs were split into two clusters, such that 71.2% of dogs belonged to dog cluster 1 (using a Q value cut-off of 0.85 for comparison), 13.1% belonged to dog cluster 2, and 15.7% dogs were split between these two clusters (Supplementary Tables file: Table 3). Therefore, *K* = 3 was considered the best fit to the data. The results from PCA analyses (Fig. 1B) were consistent with the sNMF results for *K* = 3. Red fox, raccoon dog, and golden jackal genotypes were grouped together in the first two components, clearly separated from wolf and dog genotype clusters. Although wolf and dog genotype clusters were closer together, they remained distinctive groups. The RUBIAS reference population performance tests showed 100% correct assignment to the reference populations, suggesting robust assignment.

### Validation of sex markers

Sexing was accurate in 80% of samples with known sex. Males were successfully sexed using both types of sex-chromosomal markers (Supplementary Tables file: Table 4). The Y marker amplified successfully for all 50 male samples. However, the nine X-chromosomal markers showed ambiguous sex assignment for one sample, while the remaining samples were correctly assigned as males. Females presented more challenges in sex determination, as 35% (13/37) of the females were completely homozygous for the nine X-chromosomal markers and were assigned as males. Notably, 11 of these 13 homozygous females belonged to the inbred Western Finnish wolf families used in pedigree analyses. Additionally, three samples showed ambiguous sex. The Y-marker did not amplify for most of the females (34/37), but there were three samples where the Y-marker was amplified, likely due to contamination. Sexing parameters were derived for the regular wolf monitoring (see below), by adjusting the sexing parameters until there were no mis-called sexes between the SNP data and the known physiological sex. In cases where sex could not be reliably determined, sex was classified as uncertain.

### MAF, HWE, LD and PID in the final panel

The average MAF from all high-MAF markers was 0.424, and the range was 0.292–0.500. None of the high-MAF loci were found to be linked or deviate from HWE. Based on the PID calculations, ten markers had enough power to distinguish unique individuals drawn randomly from a population, while 19 markers were required to identify unique individuals among siblings (Supplementary Tables file: Table 5).

### Pedigree analyses

Inbreeding coefficients calculated from the pedigree of inbred Western Finnish wolf packs varied from 0 up to 0.2 (Supplementary Material file: Fig. 2). The elevated inbreeding coefficient observed in this pedigree can be traced back to the pairing of a grandfather with his granddaughter, which led to a significant number of offspring (38 known pups, not all included here) between 2015 and 2021. In four subsequent generations, some of their descendants mated within the family line, contributing to the highest inbreeding coefficients in the current (2022–2023) wolf packs.

We were able to reconstruct the known pedigree (Supplementary Materials file: Fig. 2) almost completely using either SNP or microsatellite data. Both marker types encountered issues when one of the parents had a missing genotype, resulting in unknown parentage or incorrect assignment of parent-offspring relationship. Out of the 99 parent-offspring relationships used in the comparison, all were assigned correctly, except one individual (Ind ID: W_494). With SNP data, this individual had unknown parents while microsatellite data correctly assigned it to its parents. This discrepancy was most likely caused by a mistake in the genotyping process, such as a wrongly assigned sample.

Assignment probabilities to the relationship categories varied between microsatellites and SNPs (Supplementary Tables file: Table 6). When considering only relationships with assignment probabilities exceeding 0.8 in all three replicates, 90% of the SNP assignments were accepted, whereas only 52% of the microsatellite assignments were accepted.

### Regular Wolf monitoring workflow and results

Out of the 282 samples utilized in the wolf monitoring workflow, 83% met the analysis threshold: all the tissue samples (16/16), 88% of the scat samples (166/188), 66% of the urine samples (49/74), and half of the hair samples (2/4) (Supplementary Tables file: Table 7). Sex assignment was successful for 83% of the samples. Among the 166 scat samples genotyped in duplicate and passing the analysis thresholds, only eight (5%) exhibited genotype mismatch errors, resulting in an average error rate of 0.1. Considering that there are 96 markers in the panel, this equates to an error rate of 0.001 per marker. Mismatches were re-coded as missing data.

In the PCA (Fig. 2) and RUBIAS (Supplementary Tables file: Table 7) assignment, ten out of the 233 samples that passed quality filtering were identified as originating from non-target canid species—namely, the red fox, raccoon dog, and golden jackal. A subsequent RUBIAS assignment involving only dogs and wolves led to the misassignment rendering the results unreliable. One of the seven dogs (see below) was correctly classified as dog, and 14 wolves were correctly assigned as wolves. 74 wolf samples were incorrectly assigned as dogs, and the remaining 129 wolf samples and six dogs had the highest probability to be assigned to neither dog nor wolf.

**Fig. 3:**
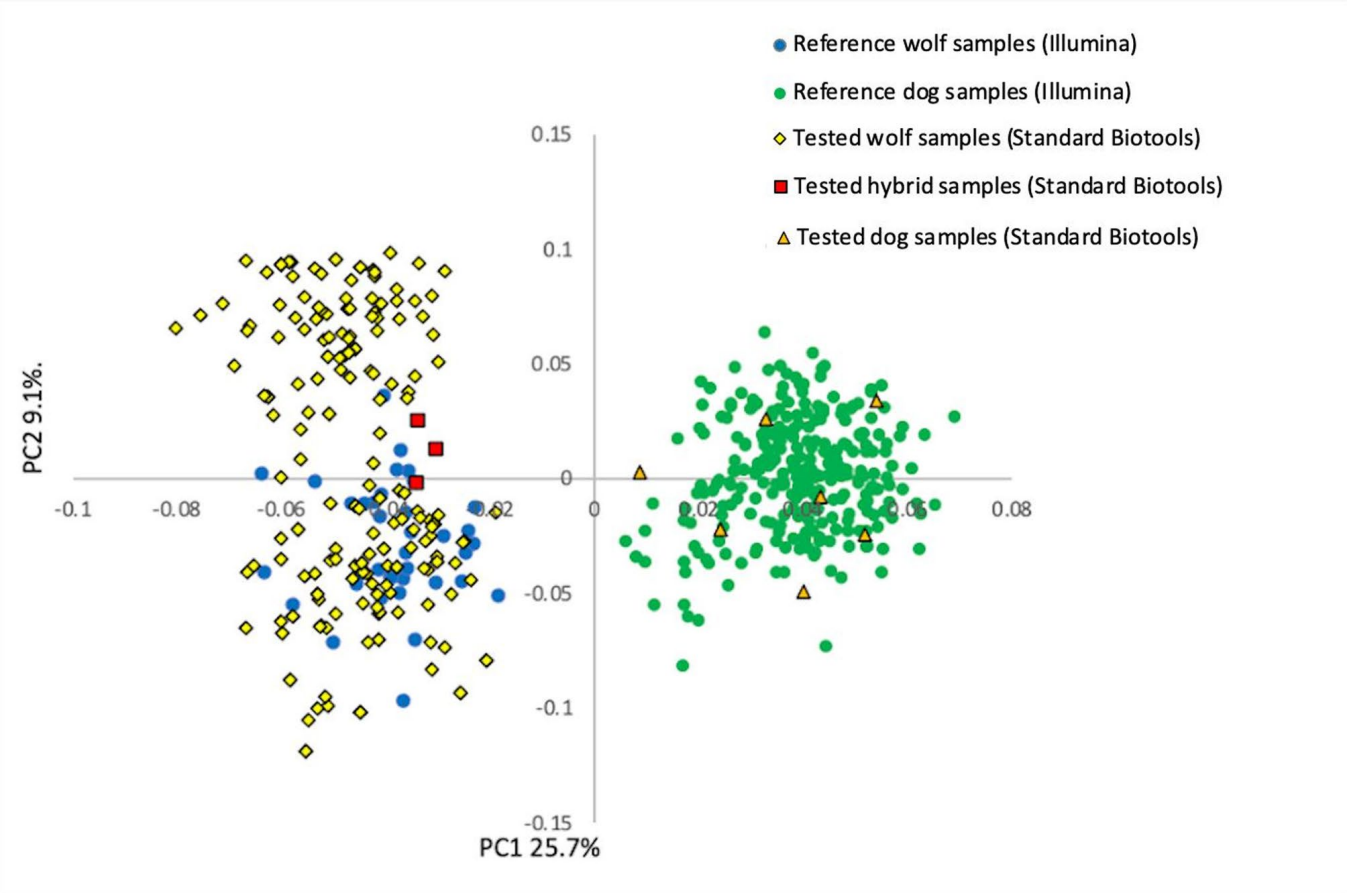
A principal component analysis (PCA) for 233 samples representing a subset of wolf monitoring data from 2022–2023, analyzed alongside reference samples of wolves and dogs and hybrids.

However, a PCA analysis focusing solely on wolves and dogs indicated the presence of seven dogs in the sample set (Fig. 3). This finding was subsequently confirmed with the wolf-dog hybrid panel^22^ in Heikkinen et al.^40^. Notably, the PCA could not distinguish hybrids from wolves. Therefore, all putative wolves, dogs and wolf-dog hybrids identified with the monitoring SNP panel are also genotyped in parallel with the 93-SNP wolf-dog hybrid panel for accurate species identification (see Supplementary Material File: supplementary protocol ).

The remaining 223 samples (including dogs and putative hybrids, Supplementary Materials file supplementary protocol) corresponded to 140 unique individuals. An allele-match analysis established a mismatch threshold of 16 SNPs, indicating that 79 SNPs were necessary for robust individual identification (Supplementary Material File: Fig. 3). The highest number of scat samples collected from the same individual was nine (Ind ID: 2023_PK_15085), with eight yielding a 100% genotypic match and one showing a 92% match due to missing data, demonstrating the high repeatability of our scat genotyping method for individual identification (Supplementary Tables file: Table 7). All unique individuals encountered during a monitoring season were subject to kin analysis, and the results were compared to kinship data from previous years (breeding pairs, known sibships) and field observation records. In particular, unique individuals with single occurrences in the data, samples close to the analysis threshold, samples with undetermined or opposite sex called from the R-script sexing, and urine samples were scrutinized to rule out cross-contamination, and samples with suspected cross-contamination were discarded.

## Discussion

This study presents the development of a custom-designed, multifunction SNP panel specifically tailored for annual genetic monitoring of wolves in Finland. It outlines our strategy to identify wolf genotypes from that of other species, sex determination, individual identification, and kinship analysis. Furthermore, it demonstrates the application of this panel in the ongoing genetic monitoring of the Finnish wolf population from 2022 to 2023 and beyond.

Our SNP panel effectively distinguished genetically distant species, such as lynx and moose, from wolves. This was evidenced by failed SNP amplification, leading to samples from these species not meeting the analysis thresholds. Similar results were observed with the 93-SNP wolf-dog hybrid SNP panel^22^, further confirming that prey species commonly consumed by wolves, such as moose, are sufficiently distantly related to canids preventing the risk of amplifying prey species from scat samples.

In contrast, samples from more closely related species such as foxes, raccoon dogs, and golden jackals passed the genotyping threshold and were clustered together in the PCA, and accurately identified in the assignment analysis. This indicates the panel’s ability to accurately differentiate between these species, despite their genetic proximity to wolves.

Wolves occasionally prey on other mammalian predators, particularly under conditions of starvation. In Estonia, raccoon dogs were estimated to constitute 10% of the wolf diet^41^, and wolves have also been observed preying on domestic dogs, albeit rarely^42^. Any potential cross-species contamination would be excluded using the same analysis protocols designed to prevent wolf-to-wolf contamination (see Supplementary Materials file Sect. 1). The panel’s ability to separate closely related species was achieved by incorporating specific autosomal markers selected to differentiate between species (dog vs. wolf, and golden jackal vs. wolf). This approach parallels previous studies in wildlife genetics, such as that of Buchalski et al.^43^, who successfully discriminated between mountain lions and bobcats using two diagnostic SNPs located in the mitochondrion. Similarly, Blåhed et al.^44^, differentiated closely related cervids using five mitochondrial SNPs, while Wehrenberg et al.^45^ discriminated among sympatric bovine species with five autosomal diagnostic markers.

The principal component analyses performed on all canids, and that on only wolves and dogs, were also successful in differentiating dogs from wolves, however the assignment analysis using only dogs and wolves failed. This was likely because the major allele frequency differences in the dataset were between foxes, raccoon dogs and golden jackals as one group, and wolves and dogs as a second group. After removing the more distant canids there was less power for assignment of the genetically close dogs and wolves. Moreover, ascertainment bias stemming from the use of the Illumina CanineHD BeadChip may have further compromised the separation of dogs and wolves with both sNMF and RUBIAS. The Illumina CanineHD BeadChip was developed to maximize polymorphism found among modern dog breeds. Funk and Kidd^26^ showed that although common variants shared across canines (including wolves, modern breed dogs, and village dogs) represent approximately 22% of the total variation among canine genomes, they account for 65% of the markers on the Illumina chip. Hence, the dogs included in the reference data have inflated genetic variation in the dataset compared to the wolves. This was evident in the sNMF analysis which showed that the wolf genetic cluster and the combined fox, raccoon dog and jackal genetic cluster were stable across *K* = 3 and *K* = 4, however, the dogs split into two more diffuse genetic clusters for *K* = 4, suggesting further substructure. Determining the most accurate *K* from assignment analyses is known to be challenging when there is unobserved ancient structure in the dataset^46^. This most likely also lead to the observed misassignment of wolves to the dog breeds using RUBIAS. It is possible that the RUBIAS analysis could be improved by defining two reference population for the dogs. This was not tested, since it does not solve the fundamental problems of ascertainment bias due to the marker selection, and because the PCA analysis nevertheless separate dogs from wolves reliably.

While the panel can separate pure dogs from wolves using PCA, it is important to note that it is intended to be used alongside a dedicated 93-SNP wolf-dog hybrid panel^22^. All new individuals identified with the monitoring SNP panel as potential wolves, dogs or wolf-dog hybrids are further genotyped with this dedicated SNP panel. By employing the 93-SNP wolf-dog hybrid panel alongside the monitoring panel, we enhance the resolution between wolves, dogs and different hybrid generations, enabling the detection of wolf backcrosses up to the third generation (fourth-generation hybrids)^22^. This comprehensive approach ensures accurate identification of dogs and hybrids and supports effective population management strategies for mitigating the risks posed by wolf-dog hybridization for the natural wolf population. Accurate hybrid identification is important in Finland since domestic wolf-dog hybrids are legislated to be nationally invasive alien species if hybridization has occurred within the last four generations (Act on managing the risks caused by alien species 704/2019; https://vieraslajit.fi/lajit/MX.5014936*).*

A crucial aspect of annual wolf monitoring is the identification of breeding units, i.e., reproducing family packs, necessitating accurate sex assignment to identify breeding females and males within a pack. Our results showed that sexing relied primarily on the Y-chromosomal marker, which exhibited 80% accuracy in the samples with known sex. The lower accuracy in determining the sex of females using X chromosomal markers was particularly severe in samples from inbred, homozygous females from Western Finland. Similarly, the SNP panel had around 80% success rate with sexing the field-collected samples. It is important to note that in Finnish wolf monitoring fieldwork, multiple samples are often collected from the same individual over the course of a monitoring year, and sometimes across multiple monitoring years. This practice increases the likelihood of accurately determining the sex of individuals, as it allows for cross-referencing and validation of sex assignment results across multiple samples and time points. As a result, the sex determination rate from field data tends to rise slightly when all samples from a monitoring season are analyzed, especially when results from multiple years are combined^40^. Sexing with the SNP panel increased the number of successfully sexed individuals by about 10% from the PCR and agarose electrophoresis method used previously (Finnish wolf monitoring reports: https://www.luke.fi/fi/luonnonvaratieto/tiedetta-ja-tietoa/susi/suden-kantaarviot-ja-seuranta#susien-kantaarviot*).* Removing one of the X-chromosome markers with a lower MAF value and instead adding another Y-chromosome marker to our current panel could increase the confidence in the sexing based on the Y-chromosome without too much impact on the sexing based on X-chromosomal markers.

Similar SNP panels developed for genetic monitoring have employed only Y-chromosomal markers for sex determination^21,44,47^. However, recent panels appear to target a combination of Y-chromosomal and X-chromosomal markers for positive confirmation of both sexes, not just males (e.g^20,43,45,48^. In our panel, the X-chromosomal markers serve an additional purpose by indicating cross-contamination, especially in urine samples collected from snow. When a territory-holding female wolf urinates, it is common for the territory-holding male to subsequently urinate over the same spot. These mixed samples often amplify well with the SNP panel, but the R-script employed for data analysis detects the conflicting sex indicators, thereby flagging these instances for further investigation. Additionally, since the included X-chromosomal markers exhibit variability, they contribute to individual identification, unlike the Y-chromosomal marker.

To effectively identify breeding units, we focused on high heterozygosity markers capable of distinguishing unique individuals and delineating kinships in relatedness analyses. Our panel included 81 high MAF SNPs for this purpose, utilizing all markers except the Y-marker for individual identification. The number of markers needed to reach PID and PIDsibs > 0.0001 was calculated to be 10 and 19, respectively, indicating the minimum number of markers required for assigning unique IDs. These numbers are similar to those reported by^19,43–45,48,49^.

However, in our analysis of the field data, with current methodology and thresholds, we have found that we can confidently resolve unique IDs with a minimum of 79 markers. This represents a fourfold difference compared to the number of markers calculated using PIDsib analysis for Finnish wolves. Ekblom et al.^20^ similarly reported a minimum number of ~ 75 markers for individual ID in wolverines, while Thavornkanlapachai et al.^48^ achieved successful individual identification in only 60% of their successfully genotyped samples using 40 loci, attributing this to stringent filtering of both loci and samples to ensure correct individual assignment. For the Finnish wolf population, factors such as inbreeding over several generations in certain areas^50^ and random missing data in scat samples likely contribute to this discrepancy. Therefore, if reliable individual identification is crucial, especially when dealing with poor-quality non-invasive samples or populations with potential inbreeding, we recommend multiplying PIDsib values by a factor of three or four to obtain a more realistic number of markers needed for individual identification.

The PID and PIDsib calculations model ideal populations and Waits et al.^36^ found that deviations from the Hardy-Weinberg Equilibrium in natural populations leads to an underestimation of the true number of microsatellite markers required for individual identification. They made the recommendation to multiply PID by a factor of three to arrive at a more realistic number. Our results suggest that the correction factor is greater for SNP markers, which may be due to the lower numbers of alleles that SNP data generate compared to microsatellites. Moreover, the allele-matching approach we employed for individual identification is based on pairwise genetic similarities and subsequent clustering, with no underlying genetic model^51^. The approach also accounts for missing data and allele errors when individual IDs are estimated, which may increase the numbers of SNPs needed, compared to the PID and PIDsib results.

We tested the SNP panel’s capability for kinship discrimination using one of the most inbred multigenerational wolf families in Southwestern Finland. Despite inbreeding coefficients reaching up to 0.2, we constructed an almost complete pedigree using the SNP panel.Microsatellite data yielded similar results in resolving the pedigree, although there was a discrepancy in assignment probabilities, with SNPs demonstrating better assignment power. Furthermore, only SNPs were used to genotype the latest generation with the highest inbreeding coefficients, hence it is uncertain how accurately microsatellites would have resolved kinships in this last cohort. Previous studies, such as Anderson and Garza^52^, have shown through simulations that 60–100 SNPs with high (0.3–0.5) MAF are generally adequate for accurate parentage assignment, and SNPs offer the advantage of lower error rates compared to microsatellites, contributing to improved assignment accuracy. Looking ahead, SNPs could play a pivotal role in constructing a reliable pedigree for the Finnish wolf population. They may be utilized in combination with microsatellite data as suggested by Giangregorio et al.^53^ to increase the analytical power and address the complexities associated with resolving kinships in populations.

Testing SNPs from the Scandinavian wolf monitoring panel revealed that the primers and protocols are indeed easily transferable between labs, but it also highlighted that the markers’ properties might not be transferable between populations (Table 1^8^). While we initially included 48 markers with high MAFs in the Scandinavian wolf population, only 21 were retained in our final SNP panel. Similarly, Giangregorio et al.^53^ found that 45 out of 85 autosomal SNPs developed for Scandinavian bears^47^ were suitable for kinship analyses in Italian Alpine bears, while only 15 were suitable for the Apennine bears. With space for only 96 markers, these types of panels can become highly biased when markers are selected from limited data for a small focal population and selected because of their particular properties in the focal population (e.g. MAF above 0.3), both of which introduce ascertainment bias^54^. Furthermore, optimizing a 96-plex primer reaction is a complex task, and some primer combinations are less impacted by the low sample quality than others when using non-invasive samples. Hence it is not guaranteed that SNPs that perform well in one panel can be seamlessly integrated into another panel.

Microarray genotyping is often more sensitive than microsatellite genotyping on non-invasive samples such as scat and hair^19,20^. When applied to field samples, we found that the SNP panel had a pass rate of about 80%, only slightly higher than the microsatellite pass rate of 77–78%^55^. However, for scat samples, the 17 microsatellites used were amplified in five different primer multiplexes, genotyped in two pool-plexes, and scat samples were always repeated from three to six times^50^ (pers. comm. Meri Lindqvist). In comparison, SNP genotyping of samples required only a single 96-plex primer pool, and scat samples were genotyped in duplicate, resulting in greater sensitivity. Our cost per sample amounted to EUR 70/ sample with StandardBiotools (in 2024) using 96.96 genotyping arrays, compared to EUR 115/sample for microsatellites (in 2020); both costs include DNA extraction, but no VAT. Subsequent genotyping using the 93-SNP wolf-dog hybrid panel amounts to another EUR 30/ sample. High quality DNA samples are selected based on the results of the monitoring panel; consequently, duplicated genotyping with the 93-SNP wolf-dog hybrid panel is rarely required. Initial and/or additional costs include buying a genotyping system or access to it via a service lab, and initial optimization costs (see also^13^). Overall, this translates to significantly lower costs and less effort in generating equal or higher-quality genotype data compared to microsatellites.

Similar to other SNP panels relying on high-MAF markers for individual identification and kinships, our panel is unsuitable for general population genetic analyses^43^. Although, for example, genetic diversity and inbreeding estimates can be calculated and tracked through time, genome-wide patterns of genetic variation are not reflected in these estimates due to the low number of markers employed and the bias towards selecting markers with high heterozygosity.

In conclusion, this study demonstrates our approach to addressing the genetic monitoring requirements for the wolf population in Finland, ranging from the SNP panel design to monitoring implementation. Additionally, the studies referenced in the discussion illustrate how other researchers have addressed their genetic monitoring needs using similar or related approaches across various taxa. While many of the cited studies, and ours, utilize microfluidic arrays for genotyping, the findings have broad applicability to anyone designing SNP panels for genetic monitoring purposes, regardless of the technology employed.

## Supplementary Information

Below is the link to the electronic supplementary material.

Supplementary Material 1

Supplementary Material 2

## Data Availability

Data are available in the Dryad repository: https://doi.org/10.5061/dryad.80gb5mm1t.
