# Supplementary material for: Development of a cost-effective, multifunctional SNP panel and analysis workflow for Wolf monitoring in Finland
Source: Sci Rep. 2025 Nov 19;15:40816. doi: 10.1038/s41598-025-24502-w (PMC12630743; doi:10.1038/s41598-025-24502-w)
Supplement: Supplementary file 2 — Supplementary Material 2 [file 41598_2025_24502_MOESM2_ESM.docx]

# Journal: Scientific Reports

# Title: Development of a cost-effective, multifunctional SNP panel and analysis workflow for wolf monitoring in Finland

Jenni Harmoinen^1,2,^ Mia Valtonen^1^, Daniel Fischer^3^, Terhi Iso-Touru^4^, Mikael Åkesson^5^, Anne-Maarit Heikkinen^2^, Katja Holmala^6^, Ilpo Kojola^7^, Elina Salmela^8,9^, Maris Hindrikson^10^, Urmas Saarma^10^, Hannes Lohi^11,12,13^, Laura Kvist^2^, Jouni Aspi^2^, Helena Johansson^4^

^1^ Wildlife Ecology Group, Natural Resources Institute Finland, Helsinki, Finland; ^2^ Ecology and Genetics Research Unit, University of Oulu, Oulu, Finland; ^3^ Applied Statistical Methods, Natural Resources Institute Finland, Jokioinen, Finland; ^4^ Genomics and Breeding group, Natural Resources Institute Finland, Jokioinen, Finland; ^5^ Grimsö Wildlife Research Station, Department of Ecology, Swedish University of Agricultural Sciences, 739 93, Riddarhyttan, Sweden; ^6^ Natural Resources Institute Finland, Helsinki, Finland; ^7^ Wildlife Ecology Group, Natural Resources Institute Finland, Rovaniemi, Finland; ^8^ Organismal and Evolutionary Biology Research Programme (OEB), University of Helsinki, Helsinki, Finland;  ^9^ Department of Biology, University of Turku, Turku, Finland; ^10^ Department of Zoology, Institute of Ecology and Earth Sciences University of Tartu, Tartu, Estonia; ^11^ Department of Veterinary Biosciences, University of Helsinki, Helsinki, Finland; ^12^ Department of Medical and Clinical Genetics, University of Helsinki, Helsinki, Finland; ^13^ Folkhälsan Research Center, Helsinki, Finland.

Corresponding author: Helena Johansson helena.johansson@luke.fi

**Supplementary material**

**Table of contents**

1. **Supplementary Figure 1:** Average cross-entropies for 10 repeats of K 1-10 for species identification analyses conducted with sNMF function within R package LEA
2. **Supplementary Figure 2.** Known pedigree of an inbred wolf family line in southwestern Finland
3. **Supplementary Figure 3.** Parameters suggested by Allelematch for establishing individual wolf IDs based on 95 SNP markers
4. **Supplementary protocol:** Workflow in the annual wolf monitoring

**Supplementary Figure 1: Average cross-entropies for 10 repeats of K 1-10 for species identification analyses conducted with sNMF function within R package LEA**

The greatest decrease in cross-entropy was between 2 and 3 populations and the lowest cross-entropy value was 4, suggesting K=3 or 4 as the most likely number of clusters. Both K=3 and K=4 were explored. K=3 was ultimately chosen as the best fit because all dogs were collected in the same cluster (Supplementary table 3), and the results were supported by the PCA.


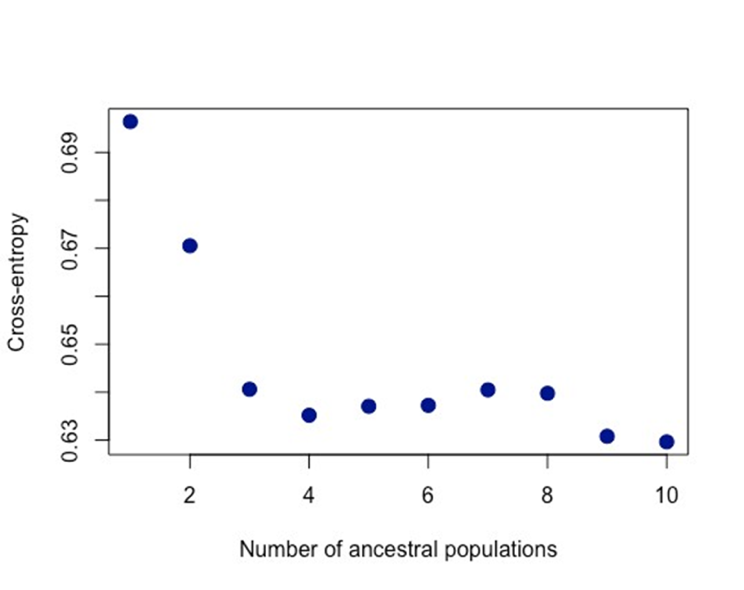


**Supplementary Figure 2. Known pedigree of an inbred wolf family line in southwestern Finland**. Pedigree-based inbreeding coefficients are marked after individual IDs. Circles represent females, rectangles represent males, and rhombuses represent individuals with unknown sex. †=no sample/genotype available, ‡=no microsatellite genotype available.


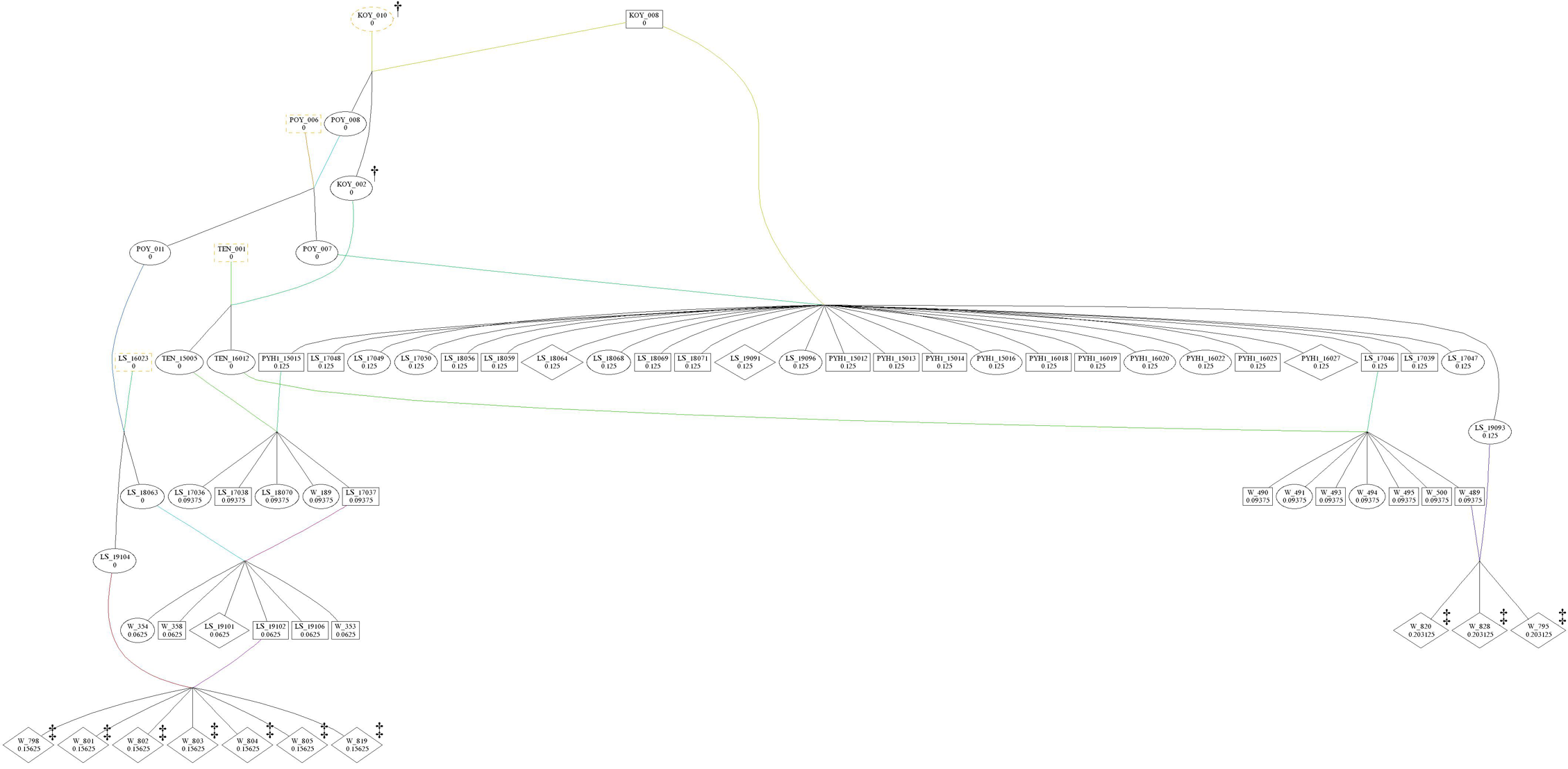


**Supplementary Figure 3. Parameters suggested by Allelematch for establishing individual wolf IDs based on 95 SNP markers**


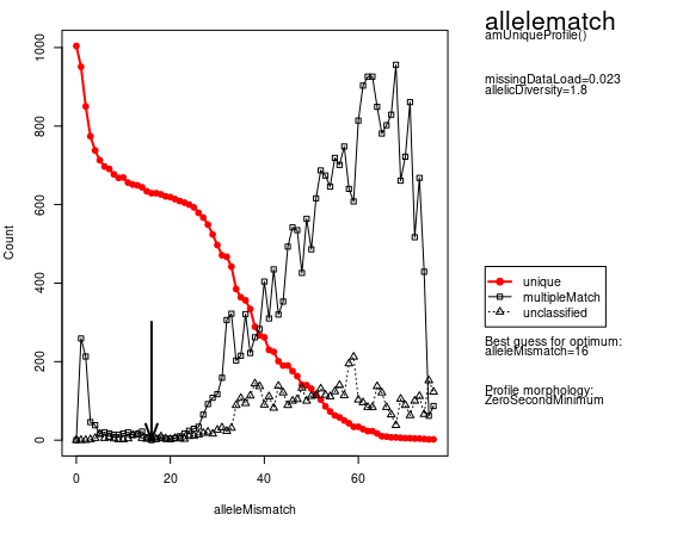


**Supplementary protocol: Workflow in the annual wolf monitoring**

**1.1. Basic filtering based on error and success rate, and sexing**

Output files (.map) from the SNP analysis software were analyzed using an edited custom set of R scripts originally adapted from Ekblom et al. [1], allowing a maximum of 19 loci with missing data for all samples and a maximum error rate of 5% when combining duplicated scat genotypes. In the script, sexing was conducted such that males had at least one successful genotype with the Y-chromosomal marker and no heterozygous genotype with X-chromosomal markers.To identify a female, the sample had to be heterozygous at a minimum of eight X-chromosomal markers and have a missing Y-chromosome genotype. The sexing parameters were derived from comparing SNP-based sexing against physiologically determined sex (see main manuscript) and adjusting the values until mis-called individuals were labeled as ‘uncertain. The output from the R scripts includes a summary file with overall success rates and success rates for species and sex markers (Supplementary table 4), as well as two sets of PLINK files, one of which includes samples that pass the specific threshold.

**1.2. Identification of wolf genotypes from non-target species**

For filtering out non-target species, the .ped file containing samples that passed the thresholds was merged with known reference samples (wolf (Illumina, Standard Biotools), dog (Illumina), fox (Standard Biotools), raccoon dog (Standard Biotools) and golden jackal (Illumina, Standard Biotools) (see Figure 1) and analysed using Principal Components Analysis (PCA) performed in PLINK [2], along with the species assignments using RUBIAS[3].

Individuals determined to be fox/raccoon dog/golden jackal were removed from further analysis, but putative wolves, dogs and hybrids between dogs and wolves were retained in the analyses until accurate species discrimination was conducted using a dedicated 93-SNP wolf-dog hybrid SNP panel[4].

**1.3. Assigning unique IDs**

Allellematch[5]was employed to identify unique multilocus genotypes among the wolves, dogs and hybrids. We utilized the optimal mismatches calculated with the function amUniqueProfiles for the entire 2022-2023 monitoring dataset, specifically a mismatch optimum of 16, a match threshold of 0.884 and a cut-height of 0.116. The X marker BICF2P1098274 exhibited high missing rates in this subset, so the analysis was performed without it.

**1.4. Cross-contamination checks and kinship analysis**

Unique IDs were checked for indications of cross-contamination. Particular attention was given to unique individuals based on single occurrences, samples close to the analysis threshold, samples where sex could not be determined by the R-script, and urine samples. The software Sequoia [6] was used for kinship analyses together with COLONY[7].

Putatively contaminated IDs were indicated by Sequoia as having genotypes too similar to another individual during an initial, integral data check in the kinship analysis process. If those highly similar individuals did not clearly assort among (often known) familial relationships from the COLONY analyses, it was considered evidence of contamination. Additionally, heterozygosities for the putatively contaminated samples and their putative relatives were calculated and compared; it is common for contaminated samples to exhibit higher heterozygosities than observed among other family members. No contamination was indicated in the samples used to demonstrate this analysis workflow.

**References**

[1] Ekblom, R., Aronsson, M., Elsner-Gearing, F., Johansson, M., Fountain, T. & Persson, J. (2021). Sample identification and pedigree reconstruction in Wolverine (*Gulo gulo*) using SNP genotyping of non-invasive samples. *Conservation Genetics Resources*, **13**, 261–274.

[2] Purcell, S., Neale, B., Todd-Brown, K., Thomas, L., Ferreira, M. A. R., Bender, D., Maller, J., Sklar, P., de Bakker, P. I. W., Daly, M. J. & Sham, P. C. (2007). PLINK: a toolset for whole-genome association and population-based linkage analysis. *American Journal of Human Genetics*, **81**.

[3] Moran, M.B & Anderson E.C. (2019). Bayesian inference from the conditional genetic stock identification model. *Canadian Journal of Fisheries and Aquatic Sciences*. **76**(4): 551-560.

[4] Harmoinen, J., von Thaden, A., Aspi, J. Kvist, L., Cocchiararo, B., Jarausch, A., Gazzola, A., Sin, T., Lohi, H., Hytönen, M. K., Kojola, I., Vik Stronen, A., Caniglia, R., Mattucci, F., Galaverni, M., Godinho, R., Ruiz-González, A., Randi, E., Muñoz-Fuentes, V. & Nowak, C. (2021) Reliable wolf-dog hybrid detection in Europe using a reduced SNP panel developed for non-invasively collected samples. *BMC Genomics*, **22**, 473.

[5] Galpern, P., Manseau, M., Hettinga, P., Smith, K., & Wilson, P. (2012). Allelematch: an R package for identifying unique multilocus genotypes where genotyping error and missing data may be present. *Molecular ecology resources*, **12**(4), 771–778.

[6] Huisman, J. (2017). Pedigree reconstruction from SNP data: parentage assignment, sibship clustering and beyond. *Molecular ecology resources*, **17**(5), 1009–1024.

[7] Jones, O. R., & Wang, J. (2010). COLONY: a program for parentage and sibship inference from multilocus genotype data. *Molecular ecology resources*, **10**(3), 551–555.
